# The immunoregulatory effect of the TREM2-agonist Sulfavant A in human allogeneic mixed lymphocyte reaction

**DOI:** 10.3389/fimmu.2023.1050113

**Published:** 2023-02-14

**Authors:** Giusi Barra, Carmela Gallo, Dalila Carbone, Marcello Ziaco, Mario Dell’Isola, Mario Affuso, Emiliano Manzo, Genoveffa Nuzzo, Laura Fioretto, Giuliana D’Ippolito, Raffaele De Palma, Angelo Fontana

**Affiliations:** ^1^ Bio−Organic Chemistry Unit, Institute of Biomolecular Chemistry, Consiglio Nazionale delle Ricerche, Pozzuoli, Italy; ^2^ Laboratory of Bio-Organic Chemistry and Chemical Biology, Department of Biology, University of Naples “Federico II”, Napoli, Italy; ^3^ Department of Internal Medicine, University of Genova, Genova, Italy

**Keywords:** small molecule, drug discovery, dendritic cells, immunoregulation, vaccine adjuvant, cancer immunotherapy, homeostasis, inflammation

## Abstract

**Introduction:**

Sulfavant A (SULF A) is a synthetic derivative of naturally occurring sulfolipids. The molecule triggers TREM2-related maturation of dendritic cells (DCs) and has shown promising adjuvant activity in a cancer vaccine model.

**Methods:**

the immunomodulatory activity of SULF A is tested in an allogeneic mixed lymphocyte reaction (MLR) assay based on monocyte-derived dendritic cells and naïve T lymphocytes from human donors. Flow cytometry multiparametric analyses and ELISA assays were performed to characterize the immune populations, T cell proliferation, and to quantify key cytokines.

**Results:**

Supplementation of 10 µg/mL SULF A to the co-cultures induced DCs to expose the costimulatory molecules ICOSL and OX40L and to reduce release of the pro-inflammatory cytokine IL-12. After 7 days of SULF A treatment, T lymphocytes proliferated more and showed increased IL-4 synthesis along with downregulation of Th1 signals such as IFNγ, T-bet and CXCR3. Consistent with these findings, naïve T cells polarized toward a regulatory phenotype with up-regulation of FOXP3 expression and IL-10 synthesis. Flow cytometry analysis also supported the priming of a CD127-/CD4+/CD25+ subpopulation positive for ICOS, the inhibitory molecule CTLA-4, and the activation marker CD69.

**Discussion:**

These results prove that SULF A can modulate DC-T cell synapse and stimulate lymphocyte proliferation and activation. In the hyperresponsive and uncontrolled context of the allogeneic MLR, the effect is associated to differentiation of regulatory T cell subsets and dampening of inflammatory signals.

## Graphical Abstract

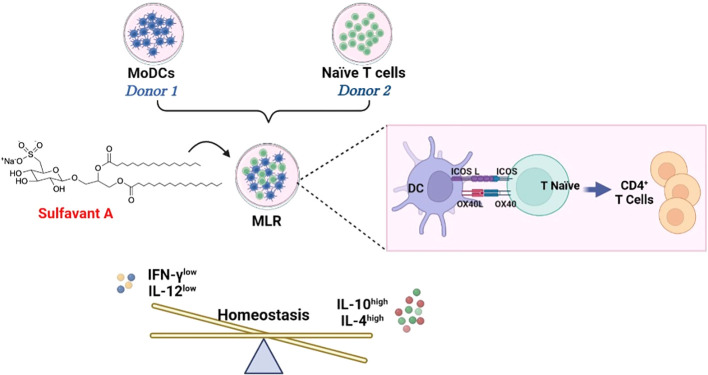

## Introduction

Dendritic cells (DCs) are innate immune cells which uniquely trigger naïve T cell activation and differentiation by providing T-cell-receptor ligands and co-stimulatory molecules (1). The interaction between DC and T cell, named the immune synapse, has a great functional plasticity that allows to modulate the activation of immunogenic or tolerogenic response (2) (3). The exchange of signals and the microenvironment established during the immune synapse helps to control the magnitude, type, and efficacy of the immune response (1). However, the mechanisms by which DCs are able to transduce the environmental stimuli and prime an effective T cell response are the result of inhibitory and stimulatory signals that are only partly understood. These signals are neither consequential nor accessory but are all part of a finely regulated bidirectional process orchestrating the dialogue between DCs and T cells. Notably, in the absence of an adequate apposition of effector and accessory molecules, T cells become anergic (1) (4). In this context, a great interest has recently arisen for new drugs capable of triggering and controlling activation and proliferation of T cells by modulating the immune synapse (5).

The mixed lymphocyte reaction (MLR) constitutes an effective tool to test activation, inhibition, or functional alteration of proliferating T cells in a model of immune microenvironment. In the classical allogeneic MLR experiments, T cells and monocyte derived DCs (MoDCs) from distinct individuals are co-cultured. The allogeneic forms of major histocompatibility complex (MHC) molecules on MoDCs stimulate proliferation and activation of T cells more strongly than conventional exposure to antigens. The technique provides a model for the study of the immune synapse (6) and, over the time, the assay has been widely employed for the preclinical tests of immunomodulatory molecules with applications in immune-oncology, autoimmunity, inflammation, vaccine development (7) (8).

Sulfavant A (SULF A) is a synthetic sulfolipid derived from naturally occurring sulfoquinovosides that are essential components of the chloroplast membranes in the photosynthetic organisms (9). The molecule primes an unconventional maturation of MoDCs through a toll like receptor (TLR)-independent mechanism leading to up-regulation of the costimulatory and MHC molecules along with hypoproduction of cytokines (10) (11) (12) (13). The molecule has been proposed as a vaccine adjuvant (EU Patent n. EP3007725B1) and despite the divergence from the inflammatory mechanisms of conventional adjuvants, retains the ability to activate immune protection in an experimental model of a prophylactic vaccine against melanoma in B16F10 mice (11). Very recently, we showed that SULF A can bind the triggering receptor expressed on myeloid cells (TREM2) which helps explain the unusual mechanism of action of the sulfolipid and the ability to trigger *in vitro* the differentiation of a homeostasis-inducing DC subpopulation that we named *homeDC* (14).

In the current study, we investigate the effect of SULF A in an experimental model of allogeneic MLR with MoDCs isolated from a human donor that were co-cultured with naïve T cells of another individual at 1:10 ratio. The sulfolipid was tested at a concentration of 10 µg/mL that corresponds to the active dose used in previous experiments to stimulate *in vitro* DC maturation (10). The aim of the study was to evaluate whether SULF A affects T cell activation and proliferation in the context of allogeneic MLR, as well as to gain more insight into the effects of SULF A on adaptive immunity and the mechanisms of immune homeostasis.

## Materials and methods

### Isolation and culture of human primary cells

Monocyte-derived dendritic cells (MoDCs) were differentiated from human peripheral blood of healthy volunteers collected from Umberto I Hospital of Nocera Inferiore, Salerno (Italy). No identifying information on the donors was retained.

After density gradient isolation of Peripheral blood mononuclear cells (PBMCs) by routine Ficoll (Ficoll Paque Plus, GE Healthcare, USA), monocytes were separated by CD14 microBeads (Miltenyi Biotec, Auburn, CA, USA). Cell purity was verified by flow cytometer (FACS) analysis using human monoclonal antibodies: CD3 Percp (SK7) (Becton Dickinson), CD14 Vioblue (REA599), and CD45 FITC (REA747) (Miltenyi Biotech, Auburn, CA, USA). The CD14+/CD3-/CD45+ cells had always a purity higher than 98%. Monocytes were cultured for 6 days in RPMI medium supplemented with 10% FBS and 1% pen/strep in the presence of IL-4 (5 ng/mL) and GM-CSF (100 ng/mL) to obtain immature dendritic cells (iMoDCs). On day 6, iMoDCs were harvested, centrifuged (10 min, 300 g) and stained with CD3 Percp (SK7) (Becton Dickinson), CD14 Vioblue (REA599), CD11c FITC (REA618), CD80 PE (2D10), CD83 APC (REA714), antibodies (Miltenyi Biotec, Auburn, CA, USA) to verify cells differentiation by flow cytometry. The CD3-/CD14-/CD83-/CD80low/CD11c+ population was always higher than 95%. Naïve T cells were obtained from different buffy coats. After PBMCs separation, cells were isolated by naïve pan T cells isolation kit (Miltenyi Biotec, Auburn, CA, USA) according to manufacturer instructions. The purity of the population was verified by FACS using the human monoclonal antibodies CD3 Percp (SK7) (Becton Dickinson), CD45RA FITC (REA562), CD45RO PE(REA611), and CCR7 PeVio770 (REA108) (Miltenyi Biotec, Auburn, CA, USA). The CD3+/CD45RA+/CD45RO-/CCR7+ population had always a purity higher than 96%.

### Allogeneic mixed lymphocyte reaction assay

MoDCs (stimulators) and naïve CD3+ T lymphocytes (responders) deriving from six different donors were cultured in a ratio of 1:10 (10.000 stimulators:100.000 responders) in round-bottomed 96-well plates with RPMI medium completed with 10% human AB serum in the presence of 10 μg/mL SULF A in PBS (MLR+SULF A). The concentration of SULF A used was established on the basis of previous dose-response experiments (10) (14). Untreated co-cultures were used as control (MLR). For the analysis of MoDCs, co-cultures were harvested and analyzed by flow cytometry after 48h. The analysis was carried out by antibody staining with CD86 PEVio 615 (REA968), CD11c FITC (REA618), CD14 Vioblue (REA599), (Miltenyi Biotec, Auburn, CA, USA) ICOSL APC (2D3), and OX40L PE (11C3.1) (Sony Biotechnology). For the analysis of T cells, co-cultures were analyzed by flow cytometry after 7 days. Functional surface markers were stained by anti CD3 Percp (SK7) (Becton Dickinson), CD4 PEVio 615 (REA623) or PE (SK3) (Becton Dickinson), CD8 APCvio770 (REA734), CD25 FITC (REA570) or PE (REA570), CD127 PEvio615 (MB15-18C9), CTLA-4 PEvio770 (REA1003), CD69 APCVIO770 (REA824), (Miltenyi Biotech) OX40 FITC (ACT35), ICOS Alexa fluor 700 (C398.4A) (Biolegend). For detection of intracellular cytokines, cells were treated for 6 hours at 37°C with 10 ng/mL phorbol 12-myristate 13-acetate (PMA), 500 ng/mL ionomycin, and 10 μg/mL brefeldin A (BFA) (Sigma Aldrich, Milan, Italy) and then stained with, IL-4 APC (BD4-8) (Sony Biotechnologies), IFNγ Vioblue (REA600), TNFα APC (REA656) and IL-17 PE (CZ8-23G) (Miltenyi Biotech, Auburn, CA, USA).

### Lymphocyte proliferation assay

T cell proliferation was analyzed by flow cytometry using Cell Trace™ CFSE Cell Proliferation Kit (Thermo Fisher, Waltham, MA, USA). Naïve T cells (at least 2x10^6^) were suspended in PBS 5% AB serum and labeled with 5uM carboxyfluorescein succinimidyl ester (CFSE). Cells were washed 3 times with 5% AB serum in PBS, suspended in the co-culture medium again and an aliquot was immediately acquired by flow cytometry to set the initial fluorescence (time zero). After addition to stimulators MoDCs, dilution of the CFSE signal was measured every day to establish the experimental conditions for cell number and proliferation rate. The 7-day endpoint was selected because T cells showed a consistent proliferation rate and their number in the well did not exceed 1,5 x10^6^/mL. CFSE dilution was recorded by flow cytometry as loss of fluorescence within 7 days from the addition of 10 µg/mL of SULF A (MLR+SULF A). Untreated co-cultures were used as blank (MLR) whereas co-cultures with 1 µg/mL phytohemagglutinin (PHA) were used as a positive control of proliferation. Gating strategies are shown in **Supplementary Figure 1**. The CFSE is reported as percentage of CFSE negative cells.

### Flow cytometry

Flow cytometry was performed by FACSAriaTM (BD Bioscience) or MACSQUANT 16^®^ Analyzer (Miltenyi Biotec). For surface staining, cells were washed in PBS and stained with Viobility™ 405/520 Fixable Dye (Miltenyi Biotec, Auburn, CA, USA) for live cell discrimination. After washing in the staining buffer (SB) (2% FBS and 0.1% sodium azide in PBS), cells were incubated at 4°C with the mixture of antibodies and isotype controls according to incubation time and quantity suggested by the manufacturers. After two additional washes, cells were acquired for the FACS analysis. For the detection of intracellular cytokines, surface markers of live cells were stained as described above. Cells were then fixed and permeabilized by BD Cytofix/Cytoperm™ kit (BD Bioscience, Frankin Lake, NJ, USA) and incubated at 4°C for 20 minutes. After two washes in BD Perm/Wash™ buffer (diluted 1:10), cells were stained with the different intracellular antibodies, according to manufacturer instructions. Cells were washed in BD Perm/Wash™ and suspended in SB for the analysis. Flow cytometry was performed on 5000 events for each population of interest according to the instrument recommendations. Isotypic and FMO controls were performed to exclude nonspecific fluorescence and set the plot axis position. Control in FITC channel and back gating strategies were used to exclude cell autofluorescence.

### Real-time PCR

Total RNA was isolated using Trizol Reagent (Thermo Fisher, Waltham, MA, USA) according to manufacturer instructions. RNA quantity and purity were measured by a NanoDrop 2000 spectrophotometer (Thermo Fisher Scientific). Sample purity was checked at A260/A280 ratio between 1.80 and 2.00. Gene expression was measured by RT-qPCR by using validated primers for *T-bet, RORγT, GATA3, FOXP3, IL-10, IL-21* and *TGFβ* after 7 days from addition of SULF A to MLR test. 18S Ribosomal RNA (rRNA) was used as a housekeeping gene to normalize sample-to-sample systematic variation in RT-qPCR. ΔCt method was used to calculate the relative gene expression.

### ELISA

IL-12p70, TNFα, and IL-10 were measured in the supernatants of the co-cultures at 48h and 7 days using commercially available kits (Thermo Fisher Scientific, Waltham, MA, USA) following the manufacturer instructions. Each sample was tested in duplicate and the colorimetric reaction (absorbance at 450 nm) was quantified by EZ Read 2000 (Biochrom Ltd, Harvard bioscience) spectrophotometer. Absorbance was converted to pg/mL according to the standard curve generated with a five-parameter logistic curve fit.

### Statistical analysis

Gates between 0.5 and 1% positive events were set for isotype controls in flow cytometry. Flow Jo v10 (BD Biosciences) or MACS Quantify (Miltenyi Biotec) software were used for the measurements. Statistical analysis between the mean values for two groups were performed by non-parametric (two-sample) T test. The paired version of the test was used when replicates were matched in the two conditions. A *P* value less than 0.05 was considered statistically significant. Graphics were drawn by GraphPad Prism 8 (GraphPad Software, San Diego California, USA).

## Results

### Effect of SULF A on T cell proliferation in the MLR assay

**Figure 1:**
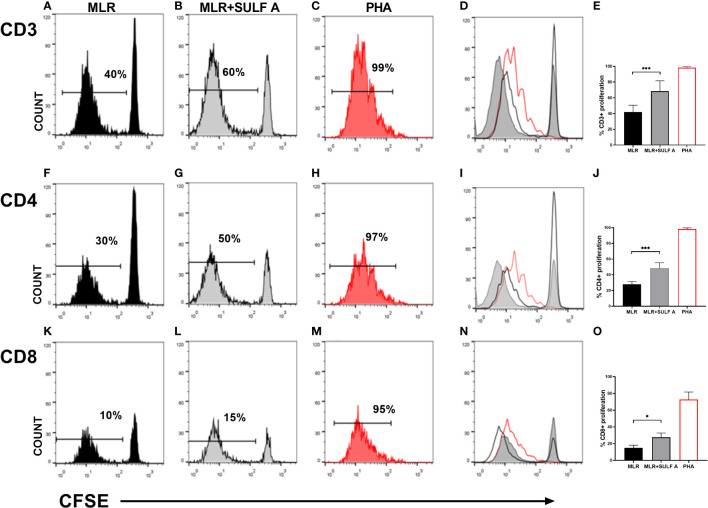
T cells proliferation induced by SULF A. Naïve T cells proliferation was assessed by CFSE dilution after 7 days of co-culture. The analysis was performed on 5000 live/T CD3+ cells. Histogram plots relative to one representative experiment are shown for CD3+ panels **(A–D)**, for CD4+ panels **(F–I)** and for T CD8+ panels **(K-N)**. Numbers in the plots indicate the percentage of proliferating cells. Panels **(D)** (T CD3+), **(I)** (T CD4+), **(N)** (T CD8+) show the overlay of the three experimental conditions for each subset. The bar graphs **(E, J, O)** represent the mean of 6 experiments. Gate strategy is reported in **Supplementary Figure 1**. MLR = untreated cocultures; MLR+SULF A = cocultures treated with 10 *μg*/mL SULF A; PHA= cocultures treated with 1 µg/mL phytohemagglutinin (positive control). Statistical analysis was performed by one-way Anova non parametric test. *P < 0.05; ***P < 0.001.

DCs are potent inducers of lymphocytes activation in allogeneic MLR. The expression of high levels of MHC and the exposition of costimulatory molecules, make them particularly suitable to elicit a strong T cell response (6). Starting from this assumption, we used the MLR model to study the effect of SULF A on naïve T lymphocytes proliferation. The MLR condition led to proliferation of about 40% of T CD3+ population after 7 days. The addition of SULF A to the co-cultures was associated to a further increase of 20%, overall reaching a mean proliferation of 60% (**Figures 1A–E**). In order to investigate whether this activation concerned T helper or cytotoxic T cells, we analysed CFSE dilution in T CD4+ and TCD8+ subsets according to the gate strategy reported in **Supplementary Figure 1**. The T CD4+ subset showed a basal proliferation of 30% reaching 50% when SULF A was added to the co-culture (**Figures 1F-J**). Slight changes were registered for the T CD8+ subset for which the baseline proliferation of 10% increased by only 5% with the addition of the sulfolipid (**Figures 1K–O**). Minor differences of proliferation were observed among the three compartments when naïve T cells were cultured in the presence of antiCD3/antiCD28 beads used as stimulus (not shown).

### DC phenotype in the MLR assay after addition of SULF A

**Figure 2:**
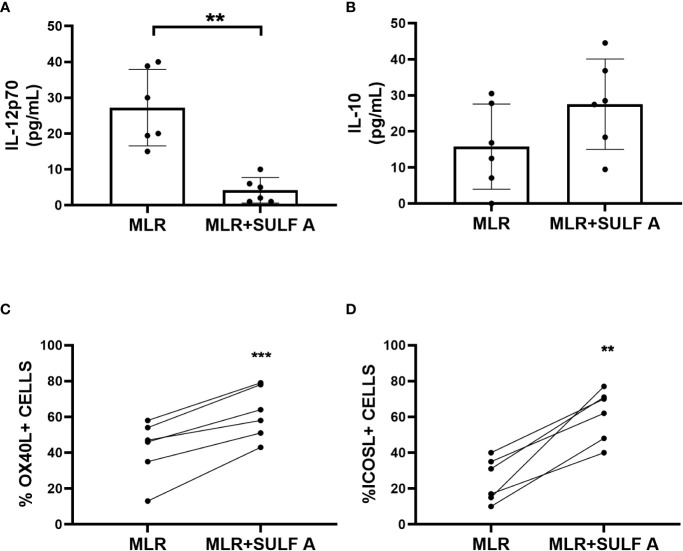
Response of cultured MoDCs with naïve T lymphocytes 48h after addition of SULF A. **(A–B)** Levels of cytokines IL-12 and IL-10 in supernatants measured by ELISA assay; **(C–D)** Surface expression of the costimulatory molecules OXO40L and ICOSL of live CD11c+/CD3- DCs measured by FACS. The gating strategy used for these markers is reported in **Supplementary Figure 3**. MLR= untreated co-cultures; MLR+SULF A= co-cultures treated with 10 *μg*/mL SULF A. Statistical analysis (n = 6) was performed by paired non parametric two tailed T test. *P < 0.05; ***P < 0.001.

To better understand the effect of SULF A on T cell response developing in the MLR context, we decided to study cytokine production along with DC phenotype. After 48 h from the treatment with SULF A, we observed that IL-12, a prototypic proinflammatory cytokine, was significantly reduced. The levels of IL-10, the main cytokine involved in the down-regulation of inflammatory processes, increased but did not reach formal statistical significance (*P* =0.0625) (**Figures 2A–B** **)**; no change was detectable for the TNFα production (**Supplementary Figure 2**). According to the gating strategy described in **Supplementary Figure 3**, flow cytometry showed upregulation of the surface expression of the OX40L and ICOSL of DCs after SULF A addition to the co-cultures (**Figures 2C–D**). OX40L and ICOSL belong to the group of costimulatory molecules which are not constitutively expressed by resting DCs but are secondary exposed on the cell surface especially in the course of tolerogenic or regulatory immune response (1) (15) (16). The binding of OX40L with OX40 promotes T cells autocrine production of IL-4 and Th2 polarization (16). Molecules able to induce OX40L upregulation on DCs are of interest for clinical treatments of atopic dermatitis and asthma (17) (18). ICOSL is mainly involved in follicular T cells polarization, adaptive tolerance, and the maintenance of effector/regulatory T cells balance (19) (20). In experimental models of allergic encephalomyelitis (EAE) and autoimmune type 1 diabetes, blockade of ICOS/ICOSL is associated to severe worsening of the diseases (21).

### Gene and protein expression by CD4^+^ T cells in the presence of SULF A

**Figure 3:**
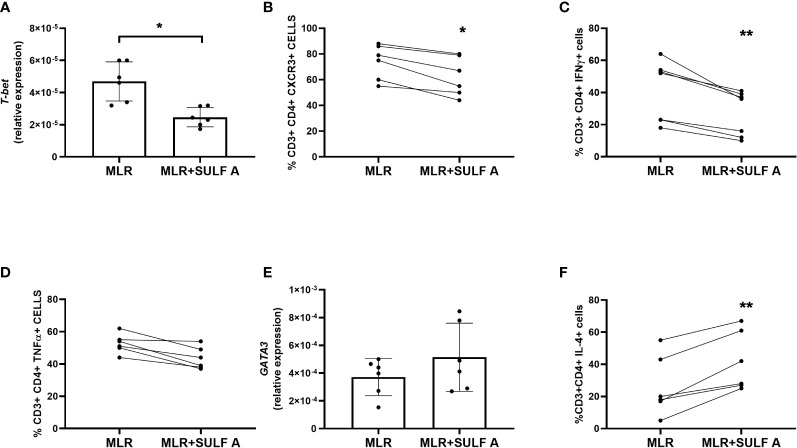
Effect of SULF A on Th1 and Th2 markers in the allogeneic MLR experiment. **(A)** RNA Expression of the transcription factor *T-bet* measured by qPCR after 7 day from the addition of SULF A to the co-cultures; **(B–D)** Surface expression of CXCR3, intracellular levels of IFNγ and TNFα measured by FACS; **(E)** RNA expression of the transcription factor GATA-3 measured by qPCR; **(F)** Intracellular level of IL-4 measured by FACS. All qPCR results (mean ± s.d) were normalized to 18S mRNA and analyzed by ΔCt method. Flow cytometry was performed on live CD3+/CD4+ cells (5000 events acquired) according to the gating strategy reported in **Supplementary Figure 4**. The intracellular cytokines were detected after a treatment of 6 hours with PMA, ionomycin and BFA. MLR = untreated co-cultures; MLR+SULF A = co-cultures treated with 10 *μg*/*mL* SULF **(A)** Statistical analysis (n = 6) was performed by paired non parametric Two-tailed T-test. *P < 0.05; **P < 0.01.

In order to characterize priming of naïve CD4+ T cell, we considered the effect of SULF A on the hallmarks of Th1 and Th2 response in co-cultures at day 7. SULF A significantly downregulated the gene expression of the transcription factor *T-bet* (**Figure 3A**) and surface expression of CXCR3 marker (**Figure 3B**), while gating analysis of flow cytometry data (**Supplementary** **Figure 4**) revealed a consistent reduction of the intracellular level of IFNγ (**Figure 3C**) but no change of TNFα (P=0.062) (**Figure 3D**). In agreement with the down-regulated production of IL-12 at 48h, these data supported the attenuation of Th1 differentiation by SULF A. Pro-inflammatory markers related to Th17 response, such as IL-17 (**Supplementary** **Figure 4**) or the transcription factor *RORγT* (**Supplementary Figure 5A**), as well as the pleiotropic cytokine *TGFβ* (**Supplementary Figure 5**) were unaffected in the allogeneic MLR and were unresponsive to SULF A addition. On the other hand, the sulfolipid increased the expression of the transcription factor *GATA3* (**Figure 3E**) and significantly increased the intracellular production of IL-4 (**Figure 3F**), both signature of type 2 immunity. Expression of *IL-21*, also implicated in the development of humoral responses, responses was found up-regulated (**Supplementary Figure 5C**) IL-4 is as multifunctional, immunoregulatory cytokine mostly associated with the suppression of pro-inflammatory signals, the dampening of the excessive immune response, and the stimulation of tissue repair and homeostasis (22).

**Figure 4:**
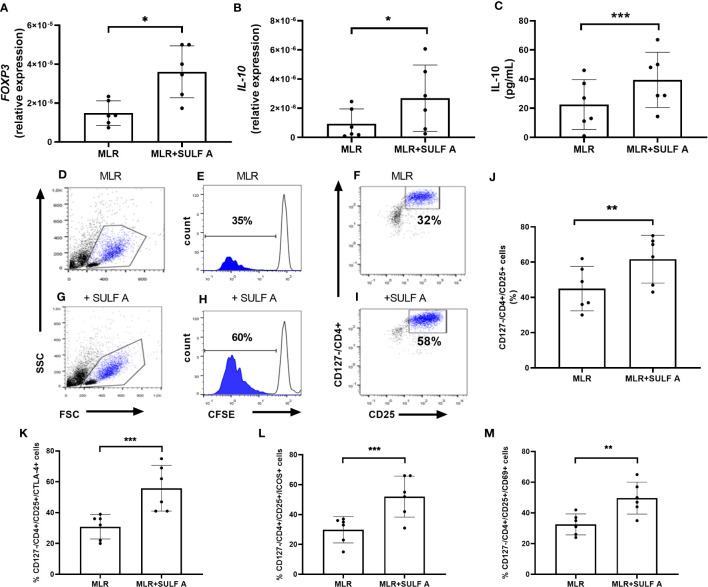
SULF A-dependent expansion of T cells in the allogeneic MLR experiment. RNA Expression of *FOXP3* **(A)** and IL-10 **(B)** measured by qPCR. Results were normalized to 18S mRNA and analyzed by ΔCt method. Values on y-axis represent the relative expression of mRNA levels compared to control. **(C)** IL-10 concentration in the MLR supernatants measured by ELISA assay. **(D–I)** Representative flow cytometry analysis of proliferating and CFSE-negative populations corresponding to the CD127-/CD4+/CD25+ subset. Analysis gated 5000 live cells. **(J)** SULF A-dependent expansion of CD127-/CD4+/CD25+ T cells (mean ± s.d) on 6 different experiments. The analysis was carried out acquiring 5000 live cells according to the gate strategy reported in **Supplementary Figure 5**; **(K-M)** Levels of cells positive for CTLA-4, ICOS and CD69 measured by flow cytomtery within CD127-/CD4+/CD25+ population. MLR = untreated co-cultures; MLR+SULF A = co-cultures treated with 10 *μg*/mL SULF **(A)** Statistical analysis was performed by paired non parametric two tailed T test. *P < 0.05; **P < 0.01; ***P < 0.001.

### T cell differentiation in the MLR assay after addition of SULF A

After 7 days, the co-cultures treated with SULF A showed a high expression of the transcription factor *FOXP3* fork head box Protein 3 (FoxP3) (**Figure 4A**) and a significant increase of IL-10 synthesis (**Figures 4B–C**). In these samples, flow cytometry gating of T cells (**Supplementary Figure 6**) revealed a consistent expansion of CD127-/CD4+/CD25+ subpopulation after addition of SULF A (**Figure 4D-J**). CD127 is a marker typically expressed on effector T cells, instead T CD4+ cells negative or low-level expressing this marker but positive for CD25 are generally associated to immunoregulation (23). In this population, SULF A also up-regulated the immune checkpoint receptor CTLA-4 (**Figure 4K**), and the inducible T-cell costimulatory receptor ICOS (**Figure 4L**). While CTLA-4 is constitutively expressed on FOXP3+/CD25+/CD4+Treg cells and is up-regulated in conventional T cells only after activation, ICOS is an indicator of T cell-mediated immune response. Notably, the initiation of the ICOS pathway is triggered by binding to ICOSL whose exposure is significantly increased on the DC surface 48h after the addition of SULF A to the MLR. According to these results, this population matched with the portion of proliferating T cells and were fully positive for the early activation marker CD69 (**Figure 4M**).

## Discussion

DCs are key players in activation and control of the immune response by presentation of the antigen to T cells in the context of major histocompatibility (MHC) molecules and delivery of additional input in the form of costimulatory surface ligands and cytokines (1) (4). The bidirectional DC-T cell synapse is a cornerstone of adaptive immunity and has been the subject of many studies although the DC response has received less attention than T cell activation and differentiation (24). The effective tuning of the immune synapse requires combination of stimulatory and inhibitory signals to provide adequate protection and prevent aberrant response leading to pathological progression. One of the best-known examples of these mechanisms is the interaction of the costimulatory molecule CD28 and the inhibitory counterpart CTLA-4 along with their ligands CD80 and CD86 (25) (26).

**Figure 5:**
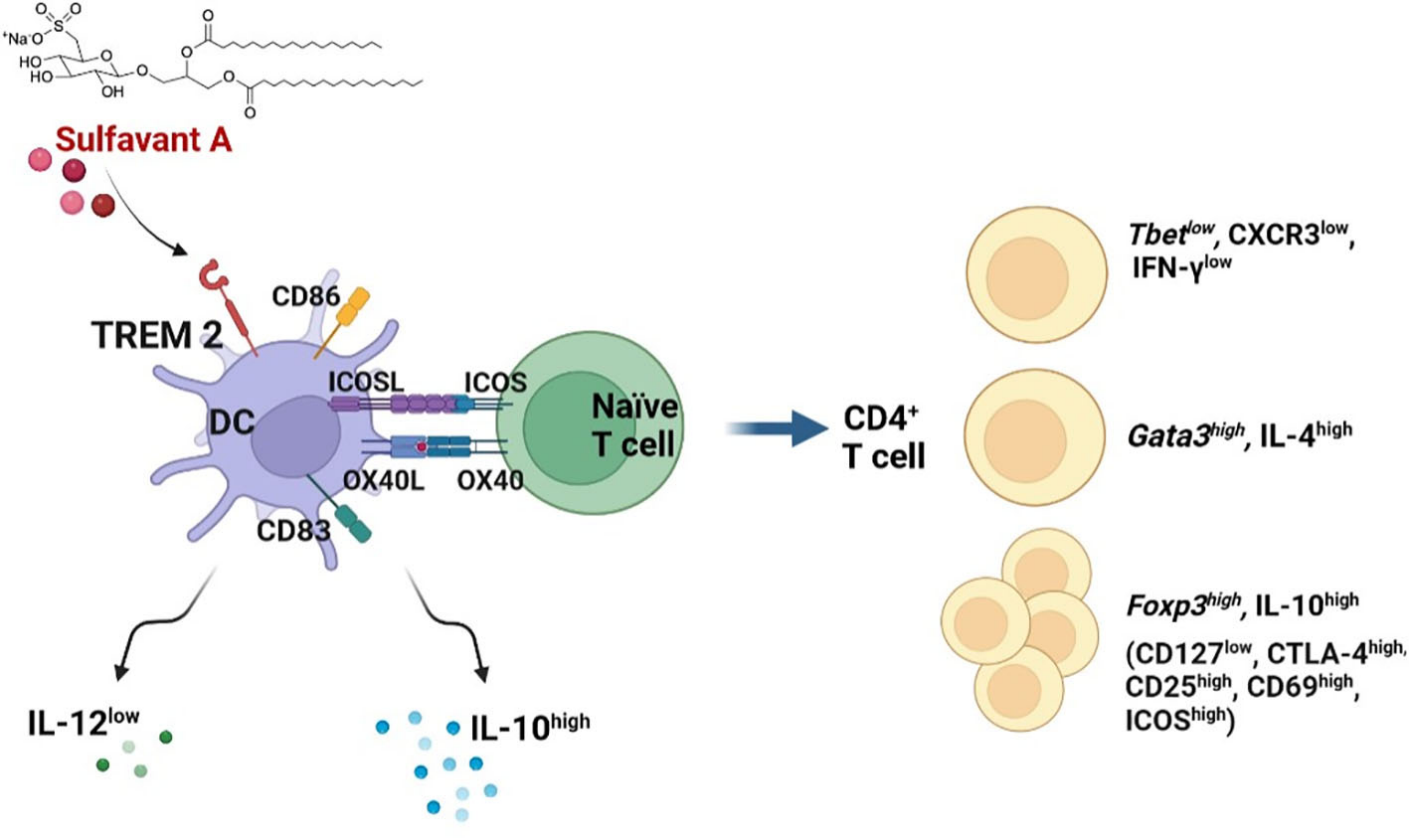
Graphic representation of the proposed mechanism of action of SULF A in MLR. After activation of DCs by TREM2, SULF A affects the stimulatory and inhibitory signalling of immune synapse. The glycolipid induces homeostasis restoration by promoting the release of IL-10, reduction of IL-12 and exposure of OX40L and ICOSL on DC membranes. The overall response is a robust T cell stimulation associated to a regulatory mechanism aimed at restoring homeostasis after the allogeneic immune reaction (image created with Biorender.com).

SULF A is a small organic molecule suitable for pharmaceutical applications due to the property of triggering an unconventional maturation of DCs and initiating an effective immune response *in vivo* (11) (14). In this study we showed that SULF A can modulate DC-T cell synapse in the context of the allogeneic MLR. According to the previous reports (14), addition of SULF A to MLR determined DC maturation after 48h with increased expression of the costimulatory molecules ICOSL and OX40L in conjunction with down-regulation of cytokine IL-12 and up-regulation of IL-10. This condition led to additional T cell proliferation and, although T CD4+ and T CD8+ subsets were both promoted, it was skewed towards T CD4+ proliferation. The whole CD4+ T cell subset also expressed high levels of *GATA3*, the main transcription factor involved in Th2 polarization (27), along with increased IL-4 production and down-regulated levels of IFNγ and CXCR3 in comparison to control. In addition to the upregulation of *FOXP3* of the CD4+ T cells, gated analysis on the proliferating subset also indicated the selection of a CD127-CD4+CD25+CD69+ population by SULF A. This finding correlates with the release of high level of IL-10 and up-regulation of ICOS and CTLA-4 expression, and suggested the priming of regulatory subpopulations functionally committed to maintain immune tolerance (23) (28). Moreover, ICOS is an activation marker that cooperates to maximize and sustain CD4+ and CD8+ T cell responses (29) but ICOS+ Tregs have stronger abilities of suppression and survival than ICOS- Tregs (30) (31) (32). It is worth noting that this response of the T cell compartment after 7 days was coherent with the early differentiation of tolerogenic DCs at 48h (**Figure 5**).

SULF A binds to TREM2, an immune receptor of the immunoglobulin superfamily, more effectively than other ligands so far reported. We put forward that the engagement of TREM2 correlates with the maturation of DCs committed to maintain homeostasis (14). The selection of this DC subset can also contribute to the regulatory response observed in the allogeneic MLR after the addition of the sulfolipid. This hypothesis agrees with the immunomodulatory properties of TREM2 that acts as a homeostasis effector in the central nervous system (33) and antagonizes the pro-inflammatory pathways originating from TLRs in DCs (34). The role of TREM2 in the immune response is still debated and could vary in relation to tissue- or organ-specific factors. However, recent studies on T CD8 + cancer infiltration indicate that the deficiency of tumor associated macrophages (TAMs) positive for TREM2 is associated with a stronger cytotoxic T cell activity and a higher responsivity to anti PD-1 immunotherapy (35) (36). In tumor microenvironment, TREM2 expressed on TAMs is also directly involved in *FOXP3* Treg cells recruitment and T CD8+ suppression, and Colonna et al., reported that anti-TREM2 mAB treatment could reduce the infiltration of immunosuppressive macrophages and expand the presence of cells expressing immunostimulatory molecules (36) (37) (38).

In conclusion, our results suggest that the immunomodulatory sulfolipid SULF A promotes a regulatory response in allogeneic MLR. Notably, the molecule triggers a negative modulation of the immune synapse by expression of co-inhibitory molecules on DCs and T cells, along with the release of cytokines, such as IL-4 and IL-10, favoring the dampening of inflammation. In the hyperresponsive and inflammatory context of the allogeneic MLR, the overall outcome is a tolerogenic effect that is likely aimed to restoration of homeostasis. However, it is possible that SULF A could elicit inhibitory or stimulatory immune responses in a context-dependent manner. This hypothesis may explain the effective activation of antigen-induced immune protection that SULF A has shown *in vivo* (11) and agrees with recent findings on the different modulation of immune cells by TREM2 and PRRs in relation to different microenvironments and physiopathological disorders (39) (40).

## Data availability statement

The original contributions presented in the study are included in the article/**Supplementary Material.** Further inquiries can be directed to the corresponding authors.

## Author contributions

GB and CG prepared and performed the experiments. DC, MA, MD supported the experimental work and analysis; GB, MA, DC, MD collected the data. GB and CG analyzed the results. GB performed the statistical analysis. EM, LF, MZ prepared SULF A; GN and Gd’I carried out the chemical analysis. RP contributed to the interpretation of the results and, together AF, conceived the study. AF planned the experiments, analyzed the results and, together with GB, drafted the manuscript. All authors contributed to the article and approved the submitted version. 
