# Supplementary material for: The immunoregulatory effect of the TREM2-agonist Sulfavant A in human allogeneic mixed lymphocyte reaction
Source: Front Immunol. 2023 Feb 14;14:1050113. doi: 10.3389/fimmu.2023.1050113 (PMC9972971; doi:10.3389/fimmu.2023.1050113)
Supplement: Supplementary file 1 [file DataSheet_1.pdf]

frontiers

Supplementary Material

The immunoregulatory effect of the TREM2-agonist Sulfavant A in human allogeneic mixed lymphocyte reaction

Giusi Barra[1†*], Carmela Gallo[1†*], Dalila Carbone[1], Marcello Ziaco[1], Mario Dell'Isola[2], Mario Affuso[2], Emiliano Manzo[1], Genoveffa Nuzzo[1], Laura Fioretto[1], Giuliana d'Ippolito[1], Raffaele De Palma[3], Angelo Fontana[1,2*]

[1]Bio-Organic Chemistry Unit, Institute of Biomolecular Chemistry, Consiglio Nazionale delle Ricerche, Via Campi Flegrei 34, 80078 Pozzuoli, Italy

[2]Laboratory of Bio-Organic Chemistry and Chemical Biology, Department of Biology, University of Naples "Federico II", Via Cupa Nuova Cinthia 21, Napoli 80126, Italy

[3]Department of Internal Medicine, University of Genova, Viale Benedetto XV 6, 16100 Genova, Italy

*To whom correspondence should be addressed: Giusi Barra, g.barra@icb.cnr.it; Carmela Gallo, carmen.gallo@icb.cnr.it; Angelo Fontana, afontana@icb.cnr.it or angelo.fontana@unina.it

[†]These authors have equally contributed.

Running title: Regulatory effect of Sulfavant A on DC-T cell crosstalk

Key-words: Small Molecule; Drug Discovery; Dendritic Cells; immunoregulation; Vaccine Adjuvant; Cancer Immunotherapy; Homeostasis; Inflammation

Supplementary Figure 1

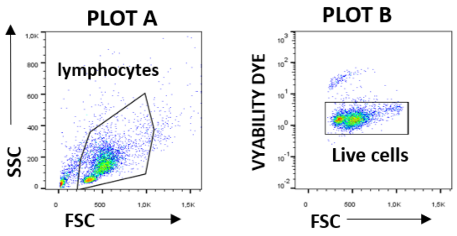

ANTIBODY	CLONE	TITRATION	PROVIDER	FACS CHANNEL
VIOBILITY FIXABLE DYE (405/452)	-	1:100	*Miltenyi biotech*	V2
CFSE FITC	-	5uM	*Thermo Fisher*	B1
CD3 PERCP	SK7	1:10	*Becton Dickinson*	B4
CD4 PEVIO615	REA623	1:50	*Miltenyi biotech*	B3
CD8 APC VIO 770	REA734	1:50	*Miltenyi Biotech*	R3

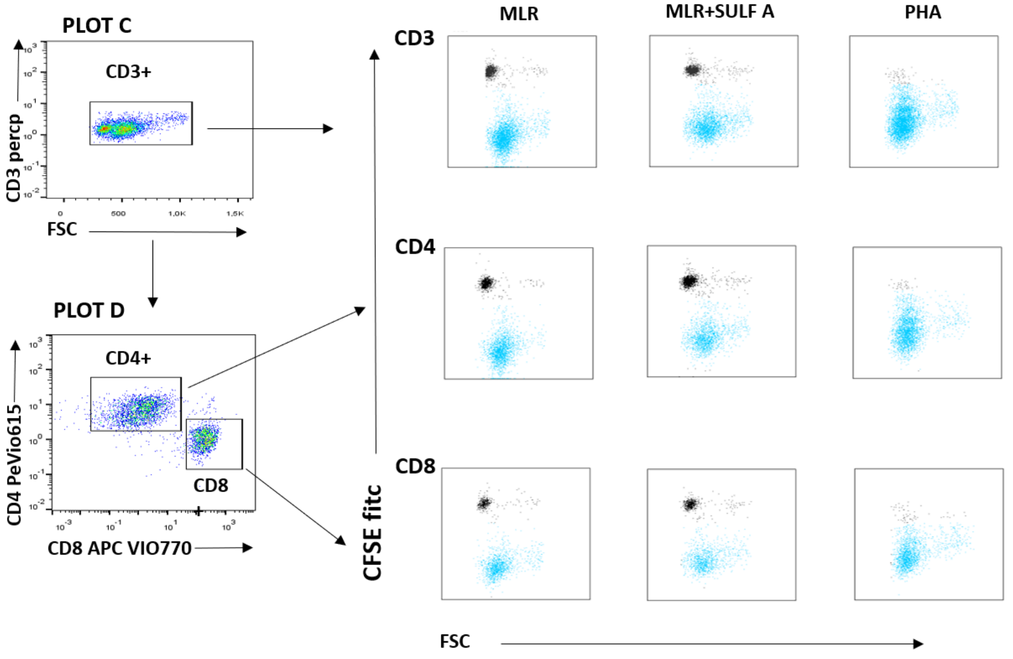

Gate strategy of CFSE analysis. Naïve T cells were stained with CFSE at T=0 and then co-cultured with DCs for the allogeneic MLR. After 7 days, cells were stained with viability dye, anti CD3 Percp, anti CD4 Pevio615 and anti CD8 APCVIO770 antibody and analyzed by flow cytometry. The physical population corresponding to lymphocytes (PLOT A) was gated and live cells were selected (PLOT B). From plot b were selected the TCD3+ (PLOT C) and gated the CD4+ and CD8+ cells (PLOT D). CD3+, CD4+ and CD8+ populations were analyzed for proliferation (light blues spots) as CFSE fluorescence dilution. One representative experiment is shown. MLR = untreated co-cultures; MLR+SULF A = co-cultures treated with 10 μg/mL SULF A; PHA= co-cultures treated with 1 μg/mL phytohemagglutinin.

Supplementary Figure 2

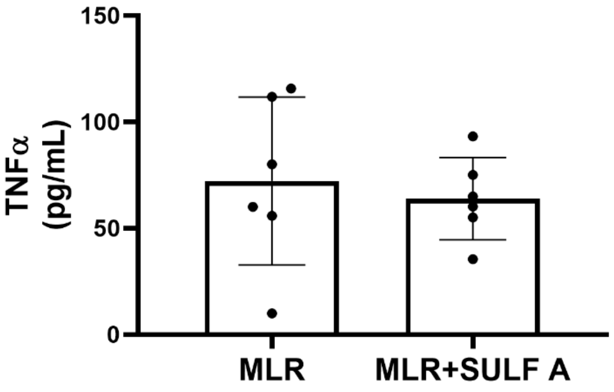

TNFα release in supernatants after 48h of co-culture. Elisa assay was conducted on the supernatants of 6 experiments. MLR = untreated co-cultures; MLR+SULF A = co-cultures treated with 10 μg/mL SULF A. Values on the y axis represent the pg/mL of TNFα.

Supplementary Figure 3

(A)

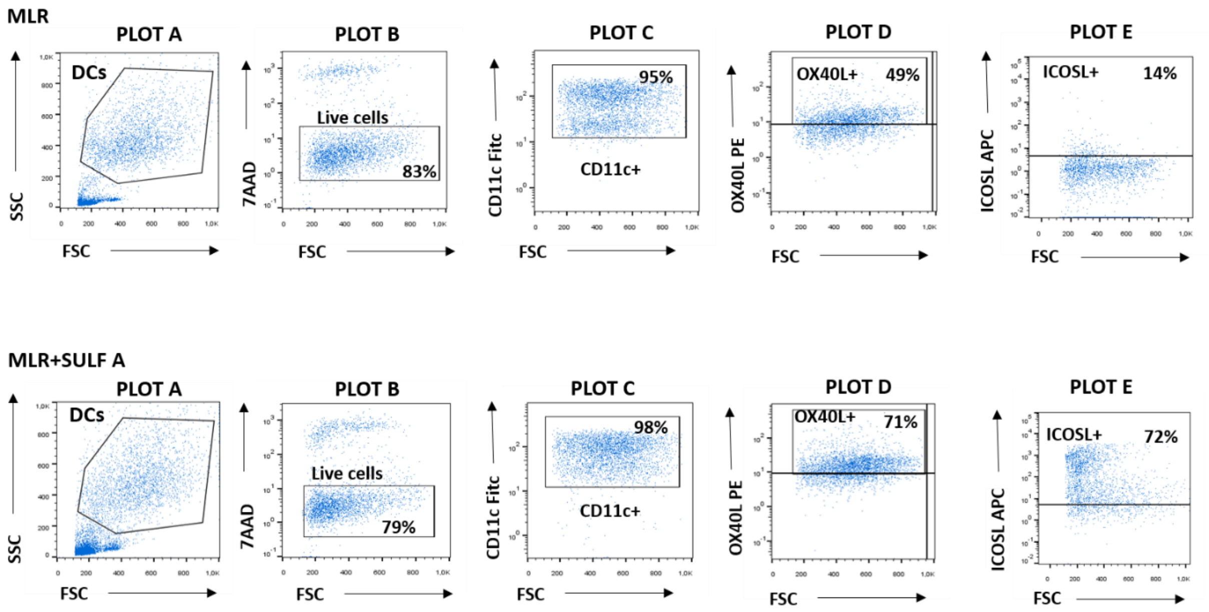

ANTIBODY	CLONE	TITRATION	PROVIDER	FACS CHANNEL
7AAD	-	1:100	*BD*	B4
CD11C FITC	REA618	1:50	*Miltenyi Biotech*	B1
OX4OL PE	11C3.1	1:10	*Sony Biotechnology*	B2
ICOSL APC	2D3	1:10	*Sony Biotechnology*	R3

(B)

	UNSTAINED CONTROL	FMO CONTROL	FULLY STAINED
FITC	-	CD11c	CD11c
PE	-	-	OX40L
APC	-	ICOSL	ICOSL

	UNSTAINED CONTROL	FMO CONTROL	FULLY STAINED
FITC	-	CD11c	CD11c
PE	-	OX40L	OX40L
APC	-	-	ICOSL

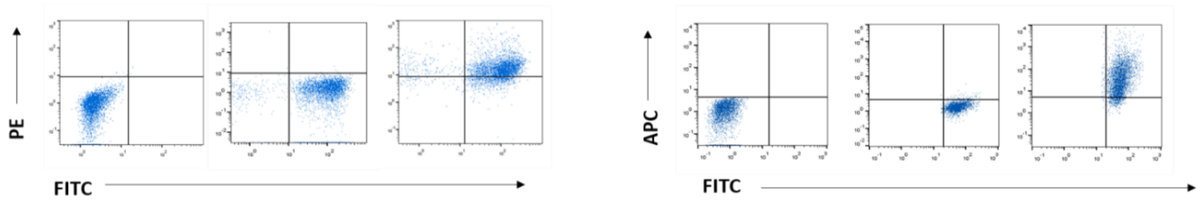

Gating strategy for OX40L+ and ICOSL+ DCs. **(A)** After the selection of the physical population corresponding to DCs (PLOT A), live cells were gated (PLOT B) and that positive for the marker CD11c (PLOT C) were selected acquiring 5000 events. Additional gating of CD11c+ cells identified CD11c+ OX40L+ (PLOT D) and Cd11c+ICOSL+ (PLOT E) sub-populations. MLR = untreated co-cultures; MLR+SULF A = co-cultures treated with 10 μg/mL Sulfavant A. **(B)** A representative experiment of Fluorescence Minus One (FMO) control for OX40L and ICOSL in the samples stained for live CD11c+ cells.

Supplementary Figure 4

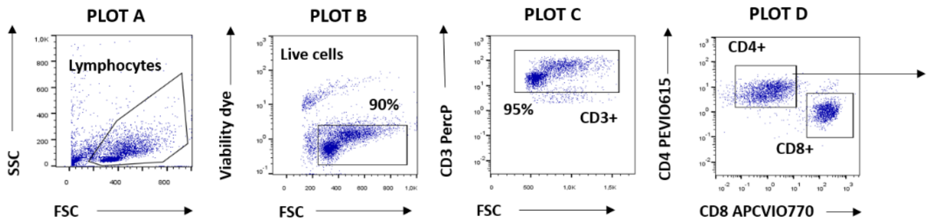

ANTIBODY	CLONE	TITRATION	PROVIDER	FACS CHANNEL
VIOBILITY FIXABLE DYE (405/452)	-	1:100	*Miltenyi biotech*	V2
CD3 PERCP	SK7	1:10	*Becton Dickinson*	B4
CD4 PEVIO615	REA623	1:50	*Miltenyi biotech*	B3
CD8 APC VIO 770	REA734	1:50	*Miltenyi Biotech*	R3
IFNG VIOBLUE	REA600	1:50	*Miltenyi Biotech*	V1
IL-4 APC	BD4-8	1:10	*Sony Biotechnology*	R1

ANTIBODY	CLONE	TITRATION	PROVIDER	FACS CHANNEL
VIOBILITY FIXABLE DYE (405/452)	-	1:100	*Miltenyi biotech*	V2
CD3 PERCP	SK7	1:10	*Becton Dickinson*	B4
CD4 PEVIO615	REA623	1:50	*Miltenyi biotech*	B3
CD8 APC VIO 770	REA734	1:50	*Miltenyi Biotech*	R3
TNFa APC	REA656	1:50	*Miltenyi Biotech*	R1
IL-17 PE	(CZ8-23G)	1:50	*Miltenyi Biotech*	B2

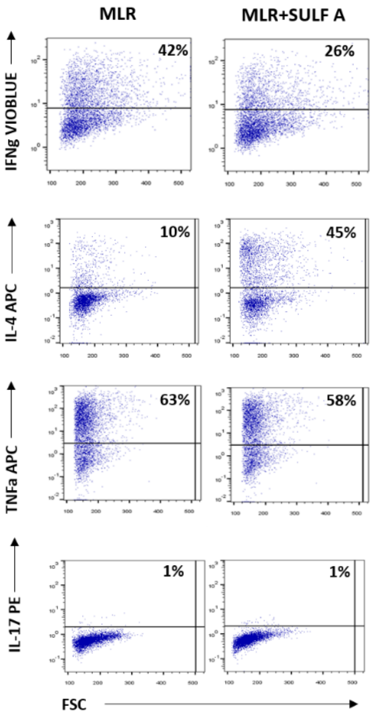

Gating strategy relative to intracellular production of IFNγ, IL-4, TNFα and Il-17 in T Cells. After 7 days of co-cultures cells were treated for 6 hours with PMA/Ionomycin and BFA. Analysis was performed by gating the physical population corresponding to lymphocytes (PLOT A). After, the selection of live cells (PLOT B), CD3+ cells were gated by acquiring 5000 events (PLOT C). From this population, CD4+ cells (PLOT D) were selected and analysed for the production of IFNγ, IL-4, TNFα and IL-17. TNFα and IL-17 were stained separately. MLR = untreated co-cultures; MLR+SULF A = co-cultures treated with 10 μg/mL SULF A.

Supplementary Figure 5

A)

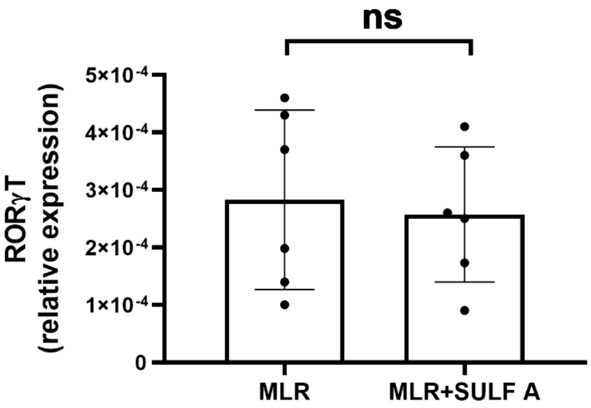

B)

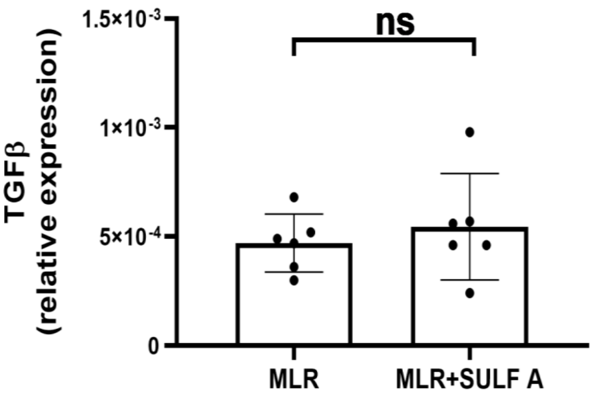

C)

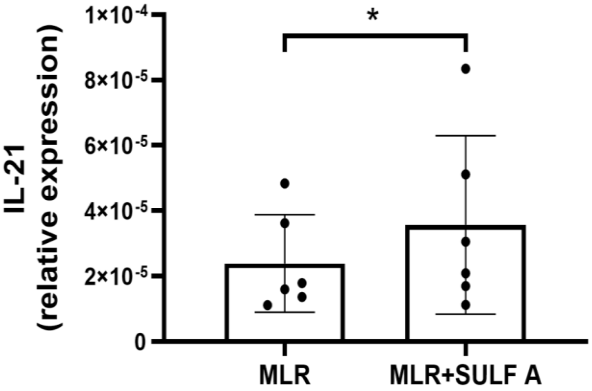

RNA Expression of the transcription factor RORγT (A), TGFβ (B) and IL-21 (**C**) measured by qPCR after 7 days from the addition of SULF A to the co-cultures. All qPCR results (mean ± s.d.) were normalized to 18S mRNA and analyzed by ΔCt method. MLR = untreated co-cultures; MLR+SULF A = co-cultures treated with 10 μg/mL SULF A. Statistical analysis (n = 6) was performed by paired non parametric Two-tailed T-test. *$P < 0.05$.

Supplementary Figure 6

(A)

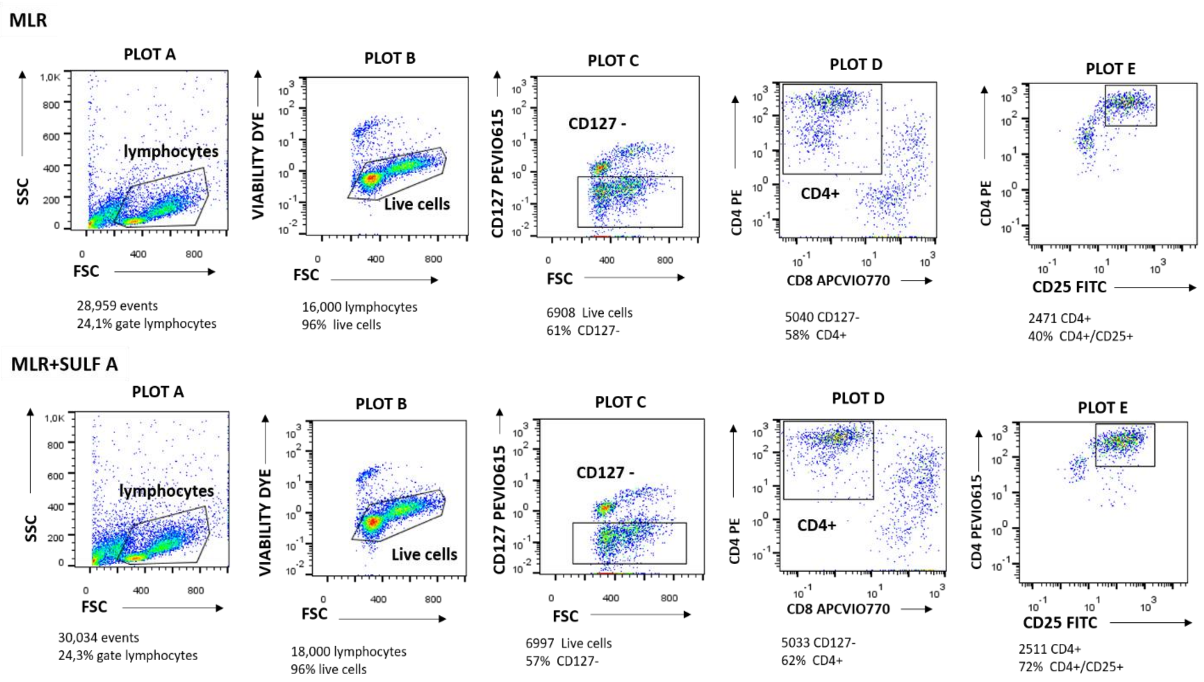

ANTIBODY	CLONE	TITRATION	PROVIDER	FACS CHANNEL
**VIOBILITY FIXABLE DYE (405/452)**	-	1:100	*Miltenyi biotech*	V2
**CD127 PEVIO615**	MB15-18C9	1:50	*Miltenyi biotech*	B3
**CD4 PE**	SK3	1:10	*Becton Dickinson*	B2
**CD8 APC VIO 770**	REA734	1:50	*Miltenyi Biotech*	R3
**CD25 FITC**	REA570	1:50	*Miltenyi Biotech*	B1

(B)

ANTIBODY	CLONE	TITRATION	PROVIDER	FACS CHANNEL
**VIOBILITY FIXABLE DYE (405/452)**	-	1:100	*Miltenyi biotech*	V2
**CFSE FITC**	-	5uM	*Thermo Fisher*	B1
**CD127 PEVIO615**	MB15-18C9	1:50	*Miltenyi biotech*	B3
**CD4 APC**	M-T466	1:50	*Miltenyi Biotech*	R1
**CD25 PE**	REA570	1:50	*Miltenyi Biotech*	B2
**CTLA-4 PEVIO770**	REA1003	1:50	*Miltenyi Biotech*	B6
**CD69 APC VIO770**	(REA824)	1:50	*Miltenyi Biotech*	R3
**ICOS ALEXA FLUOR 700**	(C398.4A)	1:100	*BIOLEGEND*	R2

Gate strategy for CD127-CD4+CD25+ subset. **(A)** After the selection of the physical population corresponding to lymphocytes (PLOT A), live cells were gated (PLOT B). Next selection was performed for the marker CD127 (PLOT C). Within cells negative for CD127, gating of the CD4+ population was performed (PLOT D) and 5000 events were acquired. Final selection identified cells double positive for CD4 and CD25 (PLOT E). MLR = untreated co-cultures; MLR+SULF A = co-cultures treated with 10 μg/mL SULF A. **(B)** Information related to antibodies used in Figure 4 of the main text.
